# Polymorphisms in *HTR2A* and *DRD4* Predispose to Smoking and Smoking Quantity

**DOI:** 10.1371/journal.pone.0170019

**Published:** 2017-01-19

**Authors:** Gloria Pérez-Rubio, Alejandra Ramírez-Venegas, Valeri Noé Díaz, Leonor García Gómez, Karina Elvira Fabián, Salvador García Carmona, Luis A. López-Flores, Enrique Ambrocio-Ortiz, Rocío Contreras Romero, Noé Alcantar-Ayala, Raúl H. Sansores, Ramcés Falfán-Valencia

**Affiliations:** 1 Laboratorio HLA, Instituto Nacional de Enfermedades Respiratorias Ismael Cosío Villegas, México City, México; 2 Departamento de Investigación en Tabaquismo y EPOC, Instituto Nacional de Enfermedades Respiratorias Ismael Cosío Villegas, México City, México; 3 Banco de sangre, Instituto Nacional de Enfermedades Respiratorias Ismael Cosío Villegas, México City, México; Yale University, UNITED STATES

## Abstract

**Background:**

Genes encoding the receptors involved in the dopaminergic and serotonergic pathways are potential candidates in the mechanisms of nicotine addiction.

**Aims:**

To identify genetic variants in the promoter regions and exons of the *DRD4* and *HTR2A* genes associated with tobacco smoking and the degree of nicotine addiction in Mexican mestizos.

**Methods:**

The study included 438 non-smokers (NS) and 1,157 current smokers, ranked based on their consumption of cigarettes per day (cpd): 574 heavy smokers (HS, >20 cpd) and 583 light smokers (LS, 1–10 cpd). Genotyping was performed for 4 and 8 single nucleotide polymorphisms (SNPs) in the *DRD4* and *HTR2A* genes, respectively.

**Results:**

The C allele of rs1800955 in *DRD4* was found to be associated with cigarette smoking in the HS *vs*. NS and LS *vs*. NS comparisons (p = 2.34E-03 and p = 1.13E-03, respectively); the association was maintained in the homozygous CC genotype (p = 5.00E-04 and p = 2.00E-04, respectively).

The T allele of rs6313 in *HTR2A* was significantly associated with cigarette smoking and a greater degree of nicotine addiction (p = 4.77E-03, OR = 1.55); the association was maintained in the homozygous genotype (TT) (p = 4.90E-03, OR = 1.96). The A allele of rs6313 was associated with cigarette smoking in the HS *vs*. NS comparison (p = 1.53E-02, OR = 1.36); the risk was nearly doubled in the homozygous AA genotype (p = 1.30E-03, OR = 1.83) compared with the heterozygous GA genotype (OR = 1.38).

**Conclusions:**

Among Mexican mestizos, the C allele of rs1800955 in the *DRD4* gene and the A allele of rs6311 in the *HTR2A* gene are associated with cigarette smoking, whereas the T allele of rs6313 in *HTR2A* is associated with cigarette smoking and the degree of nicotine addiction.

## Introduction

According to WHO, cigarette addiction is a chronic disease that evolves with relapses, and it is characterized by the inability to abstain from cigarette consumption, behavior deterioration, craving, a decline in the recognition of problems related to cigarette consumption, and dysfunctional emotional responses. The principal component of cigarettes that causes this disease is nicotine, an alkaloid that acts at the central nervous system level, causing changes in the brain of the smoker. One of the principal effects of nicotine is the alteration of certain neurotransmitter levels, including dopamine and serotonin. Therefore, the genes encoding the receptors involved in the dopaminergic and serotonergic pathways are potential candidates in the mechanisms of nicotine addiction [1, 2].

In addition to *CHRNA5*-*CHRNA3*-*CHRNB4* loci, which is the international validated loci for smoking quantity and nicotine dependence, [3, 4, 5] the most widely studied gene in the dopaminergic pathway is the dopamine receptor D4, which is encoded by the *DRD4* gene; among the most explored genetic variants are the variable number tandem repeat (VNTR), located in exon III of this gene, however, studies that evaluated single nucleotide polymorphisms (SNPs) are scarce [6, 7]. A few studies have explored the association of SNPs in the 5-hydroxytryptamine receptor 2A *(HTR2A)* gene with nicotine addiction. Some reports have identified that the rs6311 (promoter region, -1438) and rs6313 (exon) polymorphisms may be associated with a higher risk of cigarette smoking in the Caucasian population [8].

The aim of this study was to identify the genetic variants in the *DRD4* and *HTR2A* genes that are associated with cigarette smoking and a higher degree of nicotine addiction in the Mexican Mestizo population.

## Methods

### Study participants

An analytical, cross-sectional study was conducted, and it included current smokers who had smoked for ≥10 years (n = 1,157) who were recruited from the smoking cessation support clinics, which are part of the Department of Smoking Research of the Instituto Nacional de Enfermedades Respiratorias Ismael Cosio Villegas (INER) in Mexico. Subjects who were smokers were classified based on their consumption of cigarettes per day (cpd) as follows: light smokers [9, 10] (LS, n = 583), those who consumed between 1 and 10 cpd, and heavy smokers [9, 11](HS, n = 574), those who consumed ≥20 cpd. Smokers, either with symptoms (cough, phlegm, wheezing, and shortness of breath) or without symptoms, were invited for spirometry during the World Smoke-Free Day using mass media advertisements. All smokers attending our smoking cessation program were also invited for spirometry. Additionally, a comparison group consisting of non-smokers (cigarettes<100 lifetime, NS, n = 438) who were clinically healthy volunteers ≥30 years of age and without drug addictions was included.

All participants were mestizo Mexicans by birth whose parents and grandparents were born in Mexico; the participants were of both genders, were not biologically related, and were without a family history of psychiatric diseases or addictions. Subjects without psychiatric illnesses were included and were evaluated by specialized psychologists who applied the Diagnostic and Statistical Manual of Mental Disorders (DSM-IV-TR) criteria.

### Ethics statements

This study was reviewed and approved by the Bioethics and Science Committee in Research, with protocol number B15-16 and Institutional Review Board at National Institute of Respiratory Diseases (INER) Ismael Cosio Villegas.

The participants were invited to join the study and were informed about the objective of the study. They then signed an informed consent letter and were provided with an assurance-of-personal-data document. Each participant was assigned an alphanumeric key with the purpose of assuring confidentiality.

### DNA extraction

Peripheral blood (15 mL) was obtained by venipuncture and was collected in a tube with EDTA as an anticoagulant for subsequent DNA extraction using a BDtract DNA isolation kit (Maxim Biotech, San Francisco, California, USA).

The blood samples were processed in the HLA laboratory of INER to obtain genomic DNA using a commercial kit (BDtract Genomic DNA Isolation Kit, Maxim Biotech, Inc. San Francisco, CA, USA). The DNA was quantified by UV spectrophotometry using a NanoDrop 2000 (Thermo Scientific, DE, USA). Contamination with organic compounds and proteins was determined by measuring the ratio absorbance at 280 nm and 260 nm. Samples were considered of good quality when the ratio was of ~1.8.

### SNPs selection

**Fig 1:**
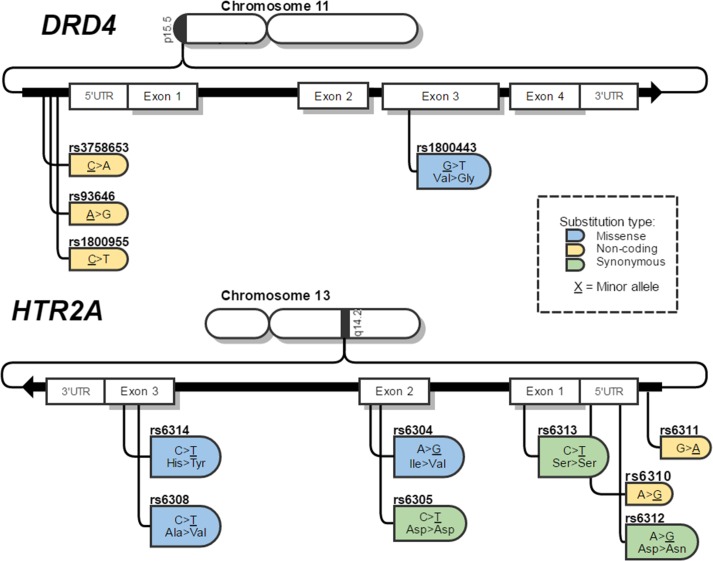
Diagram of HTR2A and DRD4 and SNPs selected for this study.

Selection of SNPs was conducted using the data from the International HapMap Project [12], and NCBI (National Center for Biotechnology Information) [13]. Only biallelic SNPs located in the regulatory regions or exons were selected; four SNPs in the *DRD4* gene and eight SNPs in the *HTR2A* gene were selected. The genetic characteristics of the SNPs are displayed in Fig 1.

### Genotyping

For genotyping by real-time PCR, 3 μL of DNA was used at a concentration of 15 ng/μL. The amplification conditions were as follows: 50°C for 2 min, 95°C for 10 min, followed by 40 cycles of 95°C for 15 seconds and a final cycle of 60°C for 1 min. The alleles and genotypes of the polymorphisms were characterized by real-time PCR (7300 Real-Time PCR System, Applied Biosystems Foster City CA, USA) via allelic discrimination using commercial TaqMan probes at 40X concentration (Applied Biosystems Foster City CA, USA). In addition, three controls without template (contamination controls) were included for each genotyping plate, and 1% of the samples included in the study were genotyped in duplicate for control allele assignment.

### Statistical analysis

The mean and the standard deviation (SD) of each variable were determined using the statistical program SPSS v.15.0 (SPSS software, IBM, New York, USA). The SNPs were analyzed with the PLINK 1.07 software [14], employing a logistic regression adjusted for age. To identify the genetic markers associated with cigarette consumption, the following comparisons were performed: HS *vs*. NS and LS *vs*. NS. The HS *vs*. LS comparison sought to establish genetic markers related to the degree of nicotine addiction. The significance values obtained in each comparison were corrected by Bonferroni’s test. The linkage disequilibrium structure and the associated haplotype generation were performed using the Haploview 4.2 software [15] with the criteria established by Gabriel and collaborators [16]. For the analysis of association by genotypes, Epidat version 3.1 software was employed [17] using simple 2x3 contingency tables, a codominant model and a 95.0% confidence level.

## Results

### Demographic variables

The demographic variables of the individuals included in the study are presented in Table 1. Age and sex were significantly different between all smokers and non-smokers; however, no statistically significant differences were found when the data were analyzed according to cpd. This behavior is consistent with epidemiological data reported in the National Survey of Addictions of Mexico (ENA, 2011), stating that male gender predominates among the Mexican smokers. The heavy smokers (HS), compared to the light smokers (LS), had been consuming cigarettes for more years and began at earlier ages.

**Table 1:** Demographic data of the population included in the study.

Variable	Smokers			NS (n = 438)	p
	All (n = 1,157)	HS (n = 574)	LS (n = 583)		
Age (years)	54 ±6	55±9	54±11	67±11	<0.001*
Sex (Male) %	47.1	51.7	42.5	17.6	<0.001**
Years of smoking	29±4	34±9	29±11		<0.001*
Cpd	17±7	24±10	7±3		<0.001*
Age at onset (years)	18±4	17±5	20±7		<0.001*

*ANOVA analysis.

**χ square test. Show mean and standard deviation.

HS, Heavy smokers; LS, Light smokers; NS, Non-smokers; Cpd, Cigarettes per day.

### Allele frequencies in mexican mestizos and other populations

Table 2 shows the minor allele frequency (MAF) for the SNPs analyzed in each study group and the average MAF in all participants (MM); data from subjects of Mexican ancestry born in Los Angeles, USA, (MEX-LA) and subjects of Caucasian (CEU) ancestry, both from the International HapMap Project, are also presented.

**Table 2:** Frequencies for minor allele in each study group and International HapMap Project population data.

Gene	SNP	MA	HS	LS	NS	All (MM)	MEX-LA	CEU
*DRD4*	rs3758653	C	0.24	0.24	0.24	0.24	0.26	0.18
	rs936461	G	0.50	0.47	0.49	0.49	NR	0.35
	rs1800955	C	0.34	0.34	0.26	0.32	NR	0.27
	rs1800443	G	0.00	0.00	0.00	0.00	NR	0.00
*HTR2A*	rs6314	T	0.05	0.04	0.04	0.04	0.06	0.06
	rs6308	T	0.00	0.00	0.00	0.00	0.02	0.00
	rs6304	G	0.00	0.00	0.00	0.00	0.01	0.00
	rs6305	T	0.02	0.01	0.01	0.01	NR	0.01
	rs6313	T	0.16	0.11	0.06	0.11	0.38	0.46
	rs6310	G	0.04	0.02	0.02	0.02	0.03	0.05
	rs6312	G	0.04	0.03	0.03	0.03	0.02	0.05
	rs6311	A	0.40	0.37	0.33	0.36	0.38	0.46

MA, Minor allele; HS, Heavy smokers; LS, Light smokers; NS, Non-smokers; MM, Mexican mestizo (our study: HS+LS+NS); MEX-LA, Mexican ancestry in Los Angeles California; CEU, Utah residents with northern and western European ancestry from the CEPH collection; NR, not reported.

Of the four SNPs in the *DRD4* gene included in the analysis, a MAF has been reported for rs3758653 alone in the MEX-LA population, which is very similar to that of the population of our study.

The MAFs of rs936461 and rs1800955 were found to be higher than those reported in the CEU population, while the MAF for rs1800443 was zero in all of the populations included in Table 3. Most of the polymorphisms in *HTR2A* have a slightly lower MAF in our population than in the MEX-LA population and especially in the CEU population; however, for rs6313, there was a significant different MAF in our NS group compared with those in the MEX-LA and especially the CEU populations.

**Table 3:** SNPs and associated alleles in the LS *vs*. NS and HS *vs*. NS comparisons.

Gene	SNP	AA	Allele frequency			HS *vs*. NS				LS *vs*. NS			
			HS	LS	NS	p*	OR	IC, 95%		p*	OR	IC, 95%	
*DRD4*	rs1800955	C	0.34	0.34	0.26	2.34E-03	1.45	1.19	1.76	1.13E-03	1.47	1.21	1.78
*HTR2A*	rs6313	T	0.16	0.11	0.05	2.05E-12	3.30	2.36	4.59	1.45E-04	2.13	1.51	3.00
	rs6311	A	0.39	0.37	0.33	1.53E-02	1.36	1.13	1.64	0.595	—	—	—

*p-values obtained by χ^2^ and after Bonferroni’s correction; AA, allele associated; HS, Heavy smokers; LS, Light smokers; NS, Non-smokers.

### Analysis of the allele, genotype, and haplotype association

Of the 12 SNPs analyzed, those that exhibited subsequent Bonferroni correction values of p <0.05 were considered to be statistically significant allelic associations (Table 3). The C allele of rs1800955 in the *DRD4* gene was found to be associated with the risk of cigarette smoking in comparisons between HS *vs*. NS and LS *vs*. NS, with similar ORs (1.45 and 1.47, respectively); this allele did not show statistically significant association with smoking in the comparison between HS *vs*. LS.

For the *HTR2A* gene, we found an association of smoking risk with the T allele of rs6313 in the comparison between LS *vs*. NS (OR = 2.13), and this association was nearly doubled (OR = 3.30) in the comparison between HS *vs*. NS. The T allele of rs6313 in the *HTR2A* gene was the only SNP associated (p = 4.77E-03) with a higher degree of nicotine addiction (OR = 1.55) in the comparison between HS *vs*. LS. The A allele of rs6311 in the *HTR2A* gene was associated with risk in the comparison between HS *vs*. NS. Genotype analysis (Table 4) also identified an association between rs1800955 in the *DRD4* gene and cigarette smoking; and in particular the homozygous genotype (CC) for the risk allele (LS *vs*. NS, OR = 2.05 and NS *vs*. HS, OR = 2.24). In the *HTR2A* gene, rs6313 presents genetic association in all three comparisons, but only with the carrier homozygote of the risk allele (T); this indicates that, in our population, that variable is associated with cigarette smoking, as well as greater nicotine addiction. In addition, the heterozygous GA and the homozygous AA genotypes of rs6311 were associated with risk in the comparison between HS *vs*. NS (OR = 1.38 and OR = 1.83, respectively). S1 Table shows the genotype frequencies for all polymorphisms tested. One SNP on DRD4 and two in HTR2A are not in Hardy-Weinberg equilibrium (HWE), data are shown in S2 Table.

**Table 4:** Associated genotypes in genes *DRD4* y *HTR2A*.

SNP	Genotype frequency (%)		p	OR	IC 95%
	**LS (n = 583)**	**NS (n = 438)**			
rs1800955					
TT	0.44 (44.56)	0.55 (55.53)		1.00	
TC	0.42 (42.14)	0.36 (36.40)	2.00E-04	1.44	1.10–1.88
CC	0.13 (13.30)	0.08 (8.06)		2.05	1.33–3.18
rs6313					
CC	0.84 (84.00)	0.90 (90.82)		1.00	
CT	0.09 (9.97)	0.07 (7.33)	3.00E-04	1.46	0.93–2.30
TT	0.06 (6.05)	0.01 (1.83)		3.58	1.64–7.80
	**HS (n = 574)**	**NS (n = 438)**			
rs1800955					
TT	0.47 (47.45)	0.55 (55.53)		1.00	
TC	0.37 (37.08)	0.36 (36.40)	5.0E-04	1.19	0.91–1.56
CC	0.15 (15.47)	0.08 (8.06)		2.24	1.46–3.44
rs6313					
CC	0.79 (79.00)	0.90 (90.82)		1.00	
CT	0.09 (9.78)	0.07 (7.33)	<0.0001	1.53	0.97–2.41
TT	0.11 (11.21)	0.01 (1.83)		7.02	3.32–14.83
rs6311					
GG	0.36 (36.20)	0.45 (45.43)		1.00	
GA	0.48 (48.39)	0.44 (44.03)	1.3E-03	1.38	1.051.80
AA	0.15 (15.41)	0.10 (10.53)		1.83	1.22–2.77
	**HS (n = 574)**	**LS (n = 583)**			
rs6313					
CC	0.79 (79.00)	0.84 (84.00)		1.00	
CT	0.09 (9.78)	0.09 (9.97)	4.9E-03	1.05	0.71–1.56
TT	0.11 (11.21)	0.06 (6.05)		1.96	1.27–3.02

p-value by linear trend test; HS, Heavy smokers; LS, Light smokers; NS, Non-smokers; NSIG, non-significant.

**Fig 2:**
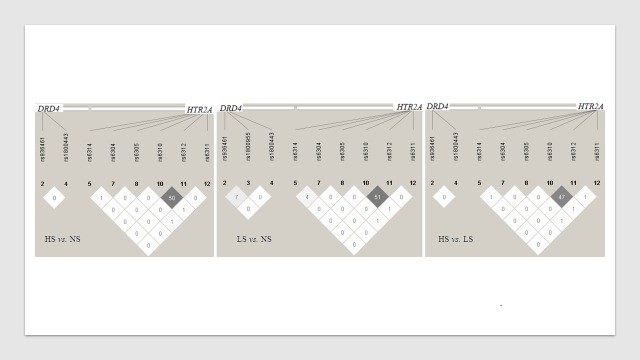
Haplotypes for *DRD4* and *HTR2A* in each comparison of the study groups. Show values of r squared in diamond. HS, Heavy smokers; LS, Light smokers; NS, Non-smokers.

Haplotype analysis revealed that high linkage disequilibrium (LD) does not exist between the polymorphisms analyzed in the *DRD4* and *HTR2A* genes, and therefore, no haplotypes are associated with the studied variables (Fig 2).

## Discussion

Nicotine addiction is a central nervous system disease that affects pathways such as the dopaminergic and serotonergic pathways in the brain. Several genetic variants have been reported to be associated with nicotine addiction, suggesting the existence of a genetic component; however, few genetic association studies in populations other than the Caucasian population have explored genetic variants in the dopaminergic and serotonergic pathways. In the *DRD4* gene, the most studied SNP is rs1800955, which is located in the promoter region (-521 C/T); in our study, in addition to this SNP, two SNPs located in the promoter (rs3758653 and rs936461) and one in an exon (rs1800443) were included.

The findings showed that only the C allele of rs1800955 is associated with cigarette smoking in the Mexican mestizo population; the association was preserved in the homozygous CC genotype. However, this SNP was not found to be associated with the degree of nicotine addiction.

Relative to its biological function, in a study in *postmortem* brains from Japanese patients with schizophrenia, Okuyama et al (1999), showed that the presence of the T allele (rs1800955), compared with the C allele, reduces the transcriptional efficiency of *DRD4* by 40% [18]; however, failed to replicate this finding using cell culture assays [19]. In other report, suggest that the presence of the T allele in rs1800955 may cause a reduction in the dopamine D4 receptors at the synapse [20] therefore; we assume that the brains of smokers carrying the C allele (risk allele) contain more D4 dopamine receptors than the brains of smokers carrying the T allele. This hypothesis would have to be proven through gene expression studies in previously genotyped smokers carrying the rs1800955 in *DRD4*. For the *HTR2A* gene, only two of the eight evaluated polymorphisms were significantly associated with risk. The A allele of rs6311 was associated with the risk of cigarette smoking in the HS *vs*. NS comparison in both heterozygous and homozygous (GA, AA) genotypes; the latter had an increased risk (OR = 1.83) compared with that of the heterozygous genotype (OR = 1.38). The T-allele of rs6313 was associated with tobacco consumption and a higher degree of nicotine addiction. The homozygous TT genotype showed associations with smoking and a greater degree of nicotine addiction. Earlier studies indicate that there is high LD (r^2^> 0.80) between rs6313 and rs6311 in the Caucasian population; however, this result was not observed in our study population (r^2^ = 0.15).

In 2011, a study among Australians reported that the TT genotype of rs6313 was associated (p = 0.011) with a higher risk of cigarette smoking (OR = 7.53, 95% CI 1.58–35.89); however, no association was found with the number of cigarettes smoked per day [8]. These results are similar to those found in our study because in the LS *vs*. NS and HS *vs*. NS comparisons, the TT genotype was associated with a greater risk of consumption (OR = 3.58 and 7.02, respectively). The OR was significant when extreme phenotypes (HS and NS) were compared; however, the OR decreased (1.96) when the degree of nicotine addiction (HS *vs*. LS) was compared.

A previous study in a Japanese population found no positive associations between smoking and rs6313 or rs6311 [21]. Possible explanations for these contradictory results include different characteristics of the study population (including age, years of cigarette smoking, etc.) and differences due to population stratification. Regarding the biological function of rs6313, in 2002, Polesskaya, in subjects with no history of mental disorders, reported that the presence of the C allele in rs6313 reduced the expression of the *HTR2A* gene by 20% compared with the T allele and that this was correlated with the protein levels [22]; however, they clarify that these findings should not be generalized because such studies may vary according to the study population. In 2006, the same group of researchers found high LD between rs6311 and rs6313 (G-C against A-T); interestingly, the G-C haplotype results in the generation of two additional CpG islands (potential methylation sites), which do not exist when the haplotype A-T is present. Additionally, in 2006, found a correlation between methylation, presence of rs6311 in the promoter region, and gene expression [23]; it is worth mentioning that methylation is usually associated with transcriptional repression and, consequently, the reduction of gene expression. However, it would be advisable to analyze both polymorphisms separately in our study due to the lack of LD between them.

Until recently the involvement of rs6313 (exon) in gene expression was unknown; however, by analysis of RNA expression in the human prefrontal cortex; which *HTR2A* is located on the antisense strand of DNA, has a novel gene structure with two transcription initiation sites (instead of only one, as described in the current databases, for example), and additional exons, which overlap with the *HTR2A-AS1* (HTR2A antisense RNA1) gene located on the sense strand. Through in silico analysis, they propose new splice sites within this genomic region and assume that rs6313 may participate in this process. *HTR2A-AS1* has three non-coding transcripts [24]; hence, Ruble *et al* propose that this is a long non-coding RNA, which regulate the transcription and processing of RNAs of nearby genes through either RNA-RNA interactions or epigenetic modifications [25].

The genetic variants in the *DRD4* and *HTR2A* genes that are associated with the risk of cigarette use may affect the rate of transcription through alternative splicing or by promoter region methylation, leading to a decrease in the expression of these receptors. Functional studies are required to test this hypothesis. Currently, our study allows us to conclude that the C allele of rs1800955 in the *DRD4* gene and the A allele of rs6311 in the *HTR2A* gene are associated with cigarette smoking, whereas the T allele of rs6313 (*HTR2A*) is associated with smoking and a higher degree of nicotine addiction in smokers of Mexican mestizo ancestry.

Some SNPs analyzed not met HWE, an example was rs6313, which showed the best association in our study. The deviations to the HWE can be due to several reasons; one of them is the structure of the population [26]. Mexican mestizo population is relatively young, consists of a mixture of different ethnic groups and European Caucasians; in previous studies [27] we have evaluated and reported population ancestry of individuals included in this study and have found that among the group of cases and controls there are not statistically significant population differences; with this knowledge, the test of HWE is necessary but is not sufficient [28,29] to think that data obtained for the rs6313 are not valid, especially because it is the genetic variable that is replicated in several populations. If this polymorphism is excluded for failing to meet HWE we would be discarding an important result that not only has been associated in other populations, it has been observed that the presence of the risk allele affects gene expression and even being regulated at the level of methylation.

A potential limitation of this study is that smokers self-reported the level of cigarette consumption. Ideally the amount of nicotine consumed should be measured from the levels of cotinine in sera. This measure is relevant because tobacco consumption (cpd) affects nicotine levels in the body. Sex was significantly different between all smokers and non-smokers; this behavior is consistent with epidemiological data reported in Mexico [30]; however, sex is an important factor that influences to nicotine addiction. In addition, our study did not consider SNPs in the genes involved in nicotine metabolism such as *CYP2A6* and *CYP2B6*. According a retrospective power analysis, these last two associations (rs6311 and rs100955) have low statistical power in our study, probably due these are relatively rare variants, which must be taken into consideration and possibly require later population validation.

## Conclusions

Among Mexican mestizos, the C allele of rs1800955 in the *DRD4* gene and the A allele of rs6311 in the *HTR2A* gene are associated with cigarette smoking, whereas the T allele of rs6313 in *HTR2A* is associated with cigarette smoking and the degree of nicotine addiction.

## Supporting Information

S1 Table Title: Genotype frequency in each study group. Legend: HS, Heavy smokers; LS, Light smokers; NS, Non-smokers.
(DOCX)

S2 Table Title: Data for Hardy-Weinberg equilibrium (HWE). (DOCX)
