# Supplementary material for: Polymorphisms in HTR2A and DRD4 Predispose to Smoking and Smoking Quantity
Source: PLoS One. 2017 Jan 19;12(1):e0170019. doi: 10.1371/journal.pone.0170019 (PMC5245876; doi:10.1371/journal.pone.0170019)
Supplement: S1 Table — Legend: HS, Heavy smokers; LS, Light smokers; NS, Non-smokers. (DOCX) [file pone.0170019.s001.docx]

Supporting table 1. Genotype frequency in each study group.

| Gene | SNP/Genotype | HS (n=574) |  | LS (n=583) |  | NS (n=438) |
| --- | --- | --- | --- | --- | --- | --- |
| Genotype Frequency (%) | | | | | | |
| *DRD4* | rs3758653 |  |  |  |  |  |
|  | TT | 54.80 |  | 56.30 |  | 54.48 |
|  | TC | 42.41 |  | 39.38 |  | 42.06 |
|  | CC | 2.79 |  | 4.32 |  | 3.45 |
|  | rs936461 |  |  |  |  |  |
|  | AA | 26.03 |  | 29.25 |  | 26.37 |
|  | AG | 47.94 |  | 46.58 |  | 48.93 |
|  | GG | 26.03 |  | 24.17 |  | 24.70 |
|  | rs1800955 |  |  |  |  |  |
|  | TT | 47.45 |  | 44.56 |  | 55.53 |
|  | TC | 37.08 |  | 42.14 |  | 36.40 |
|  | CC | 15.47 |  | 13.30 |  | 8.06 |
|  | rs1800443 |  |  |  |  |  |
|  | TT | 99.30 |  | 99.83 |  | 99.31 |
|  | TG | 0.69 |  | 0.17 |  | 0.68 |
|  | GG | 0 |  | 0 |  | 0 |
| *HTR2A* | rs6314 |  |  |  |  |  |
|  | CC | 90.58 |  | 91.38 |  | 92.50 |
|  | CT | 9.25 |  | 8.28 |  | 7.50 |
|  | TT | 0.17 |  | 0.34 |  | 0 |
|  | rs6308 |  |  |  |  |  |
|  | CC | 100.00 |  | 100.00 |  | 99.77 |
|  | CT | 0 |  | 0 |  | 0.23 |
|  | TT | 0 |  | 0 |  | 0 |
|  | rs6304 |  |  |  |  |  |
|  | AA | 99.30 |  | 99.13 |  | 99.08 |
|  | AG | 0.70 |  | 0.86 |  | 0.91 |
|  | GG | 0 |  | 0 |  | 0 |
|  | rs6305 |  |  |  |  |  |
|  | CC | 97.02 |  | 97.42 |  | 98.85 |
|  | CT | 2.79 |  | 2.58 |  | 1.14 |
|  | TT | 0.17 |  | 0 |  | 0 |
|  | rs6313 |  |  |  |  |  |
|  | CC | 79.00 |  | 84.00 |  | 90.82 |
|  | CT | 9.78 |  | 9.97 |  | 7.33 |
|  | TT | 11.21 |  | 6.05 |  | 1.83 |
|  | rs6310 |  |  |  |  |  |
|  | AA | 93.37 |  | 95.88 |  | 96.58 |
|  | AG | 6.09 |  | 4.11 |  | 3.19 |
|  | GG | 0.52 |  | 0 |  | 0.22 |
|  | rs6312 |  |  |  |  |  |
|  | AA | 92.28 |  | 94.33 |  | 94.25 |
|  | AG | 7.19 |  | 5.67 |  | 5.51 |
|  | GG | 0.53 |  | 0 |  | 0.23 |
|  | rs6311 |  |  |  |  |  |
|  | GG | 36.20 |  | 38.43 |  | 45.43 |
|  | GA | 48.39 |  | 49.56 |  | 44.03 |
|  | AA | 15.41 |  | 11.83 |  | 10.53 |

HS, Heavy smokers; LS, Light smokers; NS, Non-smokers.
