# Supplementary material for: Polymorphisms in HTR2A and DRD4 Predispose to Smoking and Smoking Quantity
Source: PLoS One. 2017 Jan 19;12(1):e0170019. doi: 10.1371/journal.pone.0170019 (PMC5245876; doi:10.1371/journal.pone.0170019)
Supplement: S2 Table — (DOCX) [file pone.0170019.s002.docx]

Supporting table 2. Data for Hardy-Weinberg equilibrium (HWE).

| Chr/Gen | SNP | p HWE |
| --- | --- | --- |
|  |  |  |
| 11/*DRD4* | rs3758653 | 0.003 |
|  | rs936461 | 0.670 |
|  | rs1800955 | 0.210 |
|  | rs1800443 | 0.942 |
| 13/*HTR2A* | rs6314 | 0.426 |
|  | rs6308 | 0.980 |
|  | rs6304 | 0.923 |
|  | rs6305 | 0.904 |
|  | rs6313 | <0.010 |
|  | rs6310 | 0.022 |
|  | rs6312 | 0.556 |
|  | rs6311 | 0.956 |
